# Medico-Chirurgical Transactions, Published by the Royal Medical and Chirurgical Society of London

**Published:** 1841-04

**Authors:** 


### Art. IX.

*Medico-Chirurgical Transactions, published by the Royal Medical and Chirurgical Society of London.* Vol. XXIII.—*London*, 1840. 8vo, pp. 436.

There is always a pleasure in the anticipation of a volume of Transactions of the Medico-Chirurgical Society, and generally the volume itself realizes the anticipated pleasure. But we cannot say quite so much of that now before us, the merit of the communications being, with two or three exceptions, but little beyond mediocrity. It is often, we may say always, too great a tax on reading-time, and patience, to be compelled to wade through pages of letter-press for some one important fact or supposed important speculation; and the impression is too often forced upon the mind by some writers, that they feel they must "make a paper," caring but little how, if so be that the "paper" be made. We have endeavoured from the volume before us to glean its few facts, after having cut away its harvest of words; and we think that a benefit would be conferred on the members if the Society subjected its volume before publication to a similar treatment.

I. A case of strangulated hernia in which the bowel was ruptured by the patient in his efforts to reduce it, is related by Mr. Travers. Beyond this fact, however, we do not see that it possesses much interest. The hernia appears to have been of the kind which the French term "hernie par engouement;" Mr. Travers terminates his communication by the relation of two cases, from which he was early disposed to decide negatively the question as to whether in the operation of hernia the ruptured parts should be returned into the abdomen without opening the sac. On this point we believe that he will find very very few surgeons at all at variance with his opinion.

II. The next paper is by Mr. George Gulliver, "*On the blood-corpuscles and pus-globules in certain animals.*" Mr. Gulliver remarks on the opinion that the pus-globules are but globules of blood deprived of their colouring matter, that his own observations have not led to any satisfactory results. It is well to remember in all these enquiries that this question is "one of more difficult solution by mere microscopic observation, than would be supposed by any one who had not specially examined the subject; for the blood-corpuscles are so singularly susceptible of modifications in form, size, and general characters, from very slight agency, that examples might readily be shown of their approximation in appearance to the globules of pus." (p. 27.)

III. Mr. James Paget has communicated some additional reasons for supposing that the *white spots upon the surface of the heart* are a result of inflammation. The fact of chief importance on which this opinion is founded, and which would constitute pericarditis a more frequent disease than it is supposed to be, is that "with these spots there almost always coincides some adhesion, by organized lymph, between adjacent parts of the pericardial membrane." This lymph sometimes consists of slender threads and granulations, and these occasionally require careful examination before they can be detected. In four cases, Mr. Paget found a band of adhesion passing from the surface of a spot to the pericardium opposite to it. He states "from the records of cases which I have examined, I find that in forty cases in which there were white spots on the heart, thirty-five have presented anormal adhesions or their remains. In five cases only were the adhesions absent, and in four cases only an adhesion was found where there were no spots." (p. 31.) The adhesions which are found in these cases are stated by Mr. Paget to be seated near the great vessels, where, in consequence of the comparatively small motion, there is every facility for their formation.

IV. The next paper consists of "*Remarks on Emphysema of the Lungs,*" by Dr. G. Budd.

"It is one of the chief objects of this paper to show that want of elasticity in the lung, in other words, absence of its natural tendency to collapse is the cause of many of the other anatomical characters of emphysema, and of most of the symptoms by which this affection is recognized. In order to show this more clearly, I shall first give a general idea of the act of breathing in the natural state. In natural breathing, when the expansion of the chest has attained its limit and inspiration is complete, a quantity of air, equal to that which has been inhaled, has been again expelled, chiefly if not wholly by means of the elasticity of the lung, which restores that organ to the volume it had before inspiration. The *muscles* and parietes of the chest do no more than follow the lung in its collapse, in every other respect they are passive; the elasticity of the lung is the main agent of inspiration. That this property of the lung is more than adequate to accomplish that action is proved by the fact, that when the chest is opened after death, and the atmospheric pressure tending to compress the lung is consequently equal to that which tends to dilate it, there ensues a still further expulsion of air from the lung effected by the residual elasticity of that organ, so that the only cause which during life prevents more complete expiration than does actually take place, is the inability of the parietes of the chest to follow the further retrocession of the lung. When therefore the lungs have their natural tendency to collapse, the amplitudeof the act of inspiration varies with the degree to which the parietes of the chest can follow them in their retrocession ; and this degree evidently depends in great measure on the obliquity of the ribs. . . . . These points being established," &c. (pp. 39-40.)

But we do not agree with Dr. Budd in supposing that the above points are established; rather, we consider them quite doubtful, if not quite wrong. Dr. Budd's proposition is, that the elasticity of the lung is the main agent in the vital act, expiration. We first of all object to any very strong argument being deduced in favour of this theory, from what is observed on the dead subject, living and dead lungs being very different organs. We next object that no allowance is made for the resilient action of the cartilaginous portions of the ribs, none for the direct action of muscles evidently calculated to diminish the size of the cavity of the chest, none for the tonic action of other muscles, which by the descent of the diaphragm in inspiration have been extended beyond their normal point of repose. The lungs are supposed to do all that is done in expiration, and for none of these various powers is any allowance made. We are much rather disposed to think that the elasticity of the lungs is a comparatively feeble power in the act of expiration ; but however this may be, it is quite clear that Dr. Budd's proposition cannot be regarded as "established."

There are some other remarks in Dr. Budd's paper, to which we must call the reader's attention. Dr. Budd notices the raised position of the ribs in emphysema of the lungs, and the little space through which they can move in inspiration, and points out the consequent modification which is effected both in the act of respiration and of coughing. It is well known that a diminished function must exist in portions of emphysematous lungs; and the pale appearance and lessened moisture of their texture, are also well known. The diminished vascularity of these parts is remarked ; and alluding to this in one case, Dr. Budd says "the coincidence of the pale, anemic condition of the pulmonary tissue with the congested state of the mucous membrane of the bronchial tubes in the same lung is worthy of observation, as showing an essential difference between bronchitis and pneumonia, a difference which has its origin in the different purpose and distribution of the pulmonary arteries." Another case is related in which this lessened vascularity of an emphysematous lung is considered as protecting a large portion of the lung from pneumonia. From some observations made by Mr. Jackson, and mentioned by Dr. Budd, it would appear highly probable that emphysema of the lungs is an hereditary complaint.

"Of twenty-eight persons affected with emphysema of the lungs, Mr. Jackson found that eighteen were the offspring of parents (father or mother) affected with the same disease, and that several of these had died in its course. In some instances the brothers and sisters of these were also emphysematous. On the other hand, of fifty persons not affected with emphysema of the lungs, three only were the offspring of emphysematous parents. I need not dwell on the interest of this fact as it regards man, but I would point it out to the breeders of horses as one of great importance, for there can be little doubt that the disease is transmitted in the same way in the horse. It is undoubtedly owing to ignorance or disregard of this fact, that broken-wind is so common, even in young horses." (pp. 49-50.)

The dilatation of the air-cells in emphysema is explained by Dr. Budd a necessary consequence of want of elasticity of the lung.

"The powerful muscles of inspiration are continually acting to dilate the chest, and thence, by virtue of atmospheric pressure, the air-cells. This agency is not counteracted as it should be by the natural elasticity of the lung, and the air-cells as well as the cavity of the chest are, in consequence, permanently dilated." (pp. 52.)

The paper terminates by some considerations on the subject of asthma, in which Dr. Budd gives his reasons for adopting the opinion that the transverse fibres of the bronchial tubes are not muscular, but that their contractions are simply the result of elasticity.

V. Dr. Burton has described "*a remarkable effect upon the human gums produced by the absorption of lead.*" We referred in our last Volume (p. 427) to this appearance as noticed by continental authors, with whose observations Dr. Burton was not acquainted when he wrote his paper. Dr. Burton regards it as a "sign which will enable physicians to establish with increased facility a precise diagnosis, in derangements of health depending on the unsuspected presence of lead; and also to obviate in many cases, the infliction of lead colic, during the treatment of other diseases by saturnine preparations." The following is Dr. Burton's description of the state of gums above referred to:

"The edges of the gums attached to the necks of two or more teeth of either jaw, were distinctly bordered by a narrow leaden-blue line, about the twentieth part of an inch in width, whilst the substance of the gum apparently retained its ordinary colour and condition, so far as could be determined by comparing the gums of these patients with those of other patients of the same class in the hospital; there was no invariable tumefaction, softening, or tenderness, about them, neither was there any peculiar fetor in the breath, nor increased salivary discharge to be observed on any of the fifty patients." (p. 66.)

We do not believe that Dr. Burton's diagnostic sign will be found so general as he imagines: we wish our readers, however, to partake of all the chances it supplies to improve their practice. It is certainly true that many diseases which fall under the physician's care seem to resemble, more than is generally supposed, the action of a poison upon the whole system, although the attempt is constantly, how often vainly, made to localize them in some particular organ.

"Cases often occur in hospital practice in which the functions of the brain and cerebral nerves are paralyzed by lead, and in which coma, vertigo, headach, amaurosis, and sometimes deafness are the most evident effects; in other instances the patients complain of articular pains resembling those of chronic rheumatism, periostitis, and secondary syphilis. In many of these cases, an inspection of the gums will assist in making a correct diagnosis. . . . . I think articular pains proceeding from the action of lead have been treated sometimes as those of chronic rheumatism, at others as those of secondary syphilis, often empirically. . . . . To obviate the opprobrium consequent on *mala praxis* originating in an erroneous diagnosis, I repeat that a careful inspection of the gums will be sufficient in most cases of illness depending on the presence of lead, to reveal immediately the origin of the ill." (pp. 74-5.)

VI. Mr. Stanley has related "*a case of disease in the posterior columns of the spinal cord.*" It is one of those puzzling cases in which, in accordance with what are supposed to be sound physiological laws, the symptoms during life have appeared to justify the conclusion that after death disease would be found in the anterior columns of the spinal cord, when after death the said disease is limited to the posterior columns. Such facts give abundant scope for speculations, which do not accord at all with our present purpose.

VII. The next communication is from Mr. Liston, "*on the arrangement of the intermediate vessels on surfaces secreting pus; with a note regarding the vascularity of articular cartilages.*" Delineations are given in this volume of the facts which Mr. Liston has described. Beneath a coating of lymph which lines the interior of abscesses, which varies in thickness, and in immediate contact with which is the purulent deposit, is a highly vascular membrane, the capillary vessels of which are most intimately interlaced and looped in their general arrangement. These vessels are regarded, with much probability, as the source of the purulent secretion. On examining a portion of an injected ulcer, the secreting vessels are found to be similarly arranged to those just described. But here they are very much and irregularly dilated, "without doubt attributable to want of support from the natural elastic covering, and in a great measure also to the affected part being often kept in a position unfavorable to the ready return of blood." Mr. Liston offers some sensible practical observations on the appearances which he has so carefully described; and speaks first of all, of "the mischievous effects of squeezing together the sides of suppurating cavities."

"By this proceeding," he says "the lymphatic coating is separated from its base; the circulation of the part is unnecessarily excited; bloody and often putrid secretion is poured out, and the general health in consequence disturbed. If a sufficient opening is made in a dependent position, the accumulated secretion is rapidly enough discharged, and the walls of the cavity come together and coalesce, through the natural elasticity and action of the parts. As regards ulcers, the paramount advantage of an elevated position of the affected part must be sufficiently obvious. The rapid disappearance of congestive swelling and of inflammation by an observance of this practice alone in many cases, must make apparent the good effects of favouring the return of blood. The larger veins, previously varicose and over-distended, become collapsed, and almost disappear. The same effect upon the varicose capillaries in the solution of continuity necessarily follows; the colour of the sore is speedily altered for the better, the painful feelings abate, and the nature of the discharge is ameliorated. Until this is the case, and so long as over-action to any degree exists, soothing and relaxing applications are advantageous; exudation of lymph and plentiful secretion of pus are thus encouraged. These are followed by mild astringents and stimulants, by which the dilated and weakened condition of the coats of the vessels may be supposed to be amended. The discharge is thus moderated, and the granulations prevented in a manner from becoming exuberant. The beneficial effects of uniform support can also be well understood." (pp. 91-2.)

We have given the preceding remarks not so much for any novelty they contain,—for, indeed, we should hope that Mr. Liston's observations respecting the rough mode of handling abscesses alluded to, were scarcely necessary in these times,—but because the description before given of the anatomical condition of abscesses and ulcers so well explains the efficacy of the best recognized modes of treatment. Mr. Liston has succeeded in demonstrating the existence of vessels in articular cartilages, and has given in a plate a sketch of the appearances presented by them.

VIII. The next paper, by Mr. Cæsar Hawkins, is "*on the diagnosis of foreign bodies in the larynx.*" Mr. H. relates an instance of this kind, and comments on the various difficulties of diagnosis presented by similar cases. The whole paper is well worthy of attentive perusal. The fact which we shall here notice is valuable as excluding certain signs usually considered as important in diagnosis, but which are here shown to be sometimes wanting when their presence might be most expected.

"In three cases in which a foreign body was fixed in the direction of the cricoid cartilage below the glottis, the severe paroxysms of coughing which are invariably looked for as evidence of the presence of a foreign body (but which really belong especially to its presence in other parts of the tube) were entirely absent in two, and were mild in the third, so as to lead the surgeon to suppose they could not arise from the entrance of the pebble, as the child asserted, and were afterwards entirely absent in the last month of her life; that even the voice was unaffected in two of the cases, although hoarse in the third case; but that in all three cases there were soreness and uneasiness in the part where the foreign body was fixed, a *noise* in inspiration or expiration, or in both, from the mechanical effect of the intruding substance, and in all the *patient asserted* that something had been *swallowed*. Where such circumstances as these are present to guide the surgeon, I consider that he is imperatively called upon to operate without delay." (p. 106.)

This opinion, we apprehend, few surgeons will be found to depart from.

IX. Connected with the same subject is a "*Case of tracheotomy*," related by Mr. Benjamin Travers, jun. From this case, which still further confirms the opinion announced in the last paper, Mr. B. Travers infers the expediency of performing tracheotomy in doubtful cases; and the inexpediency of employing any tube, whether metallic or elastic, instead of making a sufficiently capacious opening by the knife.

X. To these shorter papers succeed two essays by Dr. Marshall Hall, of a more important character. They are his second and third memoirs "*On some principles of the pathology of the nervous system*;" and as they involve questions of the greatest practical moment, we shall devote as large a space as we can afford to their consideration.

It is impossible that any great improvement in the science of physiology should be without its influence on that of pathology; and this must be especially the case with regard to the nervous system, the natural functions of which are so complex, and the morbid conditions of which are productive of so great a variety of irregular actions. Hitherto, as every practitioner knows, to say that a disease is *nervous* has almost been tantamount to a confession of ignorance as to its real nature. The discoveries of Sir C. Bell, which separated the two leading divisions of the nervous system, the *afferent* and the *efferent*, from each other, afforded the first glimpse of a more satisfactory method of viewing the subject; but, however important these were in opening a new field of investigation, we cannot but regard their immediate value in the explanation of the phenomena of disease, and in the indication of measures for its cure, as less than that of the discoveries of Dr. M. Hall. For whilst the former almost solely concerned the phenomena of sensation and the influence of mental conditions which were subjects of common observation, the latter reduce to general laws a series of phenomena previously altogether unexplained, and unveiled to us others which had been entirely unnoticed. The phenomena to which we allude are those of the *involuntary* actions of the nervous system, arising from the operation of stimuli of various kinds upon its incident trunks, and the reflexion of these stimuli in the form of motor impulses along its efferent branches; these actions being independent not only of the will but of the consciousness of the individual, and manifesting themselves as well in regard to the functions of organic as in those of animal life.

The application of Dr. M. Hall's physiological discoveries to the explanation of morbid phenomena is as yet scarcely commenced; and of this no one is more sensible than their author himself. He rightly says that scarcely any cases of nervous disease formerly observed are now of any service; and that an entirely new system of observation must henceforth be adopted, in accordance with the present state of our knowledge of the normal functions of the respective divisions of the system. The papers now before us are merely designed to indicate, from observations already made, the general course which must be pursued; the author promises ere long a more specific "Plan of Observation of Diseases of the Nervous System," which we hope will be zealously followed up by those whose opportunities afford them the means of assisting in the full developement of his discoveries.

The principal part of the first memoir is occupied with a recapitulation of the principles which may now be regarded as established, as to the relative share of the brain and the true spinal cord in the production of motion. In order that the *reflex* actions in the human being should be apparent, three conditions, Dr. M. Hall remarks, are necessary: 1. That the interference of *volition* should be removed. 2. That the *vis nervosa* and the *vis muscularis* should be unimpaired, not to say augmented. 3. That the *reflex nervous arcs* should be uninterrupted.

1. That volition interferes with some of the manifestations of the reflex function is obvious from various phenomena of sleep, and of comatose and paralytic affections. The closure of the fingers of the hand of a child in profound sleep, when the palm is touched; the closure of the eyelids on touching the eyelashes during the coma of hydrocephalus; and the excitement of spasmodic actions in limbs completely paralysed as to voluntary motion, by stimuli which have no such effect on limbs still under cerebral influence, are adduced by Dr. Hall in proof of this position. He might have strengthened it by a reference to the comparative physiology of the lower animals, in which it is pretty evident that the less the influence of the will, the more important and easily excited are the reflex actions.

2. In noticing the second principle we shall employ the term *vis nervosa* as indicating the means (whatever it be) by which a stimulus is propagated through the nervous system, so as to produce a motion. Our readers will remember that Dr. M. Hall and ourselves are at issue on this point : he taking credit for identifying the influence by which reflex actions are produced with the *vis nervosa* of Haller ; whilst we do not regard this supposed discovery as of any real importance, since nothing is actually known of the changes which take place in the nerves of voluntary motion or of those concerned in reflex action, which can enable us to affirm that they are the same or different. Now, *immediately* after a violent shock to the nervous system, the muscular irritability and the conducting power of the nervous system appear suspended, so that no reflex actions can be produced ; but at a more remote period they frequently return, and this even in an augmented degree.

3. The importance of the last principle appears self-evident ; but the particular application of it needs to be borne in mind when individual cases of nervous disease are being considered. Thus in some cases of paraplegia the reflex actions are present, in others absent. In the former the disease must be in the dorsal or cervical portions of the spinal cord, leaving its lumbar portion free to carry on the reflex actions, though its connexion with the brain is interrupted. In the latter the disease is probably within the lumbar vertebræ, involving that portion of the nervous centres through which the reflex actions of the lower extremities are produced. It is of course necessary that the whole circle should be complete, and not merely the central organs concerned in reflex actions.

In Dr. Hall's former memoir he pointed out that the muscular irritability is augmented (for a time at least) in cases of *cerebral* paralysis ; this augmentation probably resulting from its non-exhaustion by the operation of the nerves of voluntary motion. He now remarks that a corresponding facility of reaction to external stimuli is produced in tetanus, hydrophobia, and the artificial tetanus induced by strychnine, by the augmentation of the vis nervosa, so that the slightest external stimulus is sufficient to excite reflex actions in their most terrific forms. " What is remarkable is, that it is precisely the functions of the orifices and the sphincters, of the ingestors and egestors, which are most affected in these formidable diseases ; and, most of all, the larynx, the pharynx, the organs of respiration, and the rectum." It is over these organs that the spinal system has a peculiar control.

The localization of the effects of remedies is another important branch of the enquiry touched on by Dr. Hall in this memoir. " Strychnine acts upon the glottis [we should rather say that it acts on the whole cord, but sometimes has a peculiar operation on the muscles of the larynx;] cantharides upon the neck of the bladder; aloes on the rectum ; the secale cornutum on the uterus : all organs specially under the influence of the excito-motory power and reflex action of the spinal marrow." The action of cantharides upon the lower segments of the spinal cord is illustrated by the following curious case : " A young lady, aged twentyseven, had a fatty tumour within the tenth and eleventh dorsal vertebræ; it gradually, but completely, severed the spinal marrow, and induced paraplegia. The bladder lost its power of retention. On giving a dose of tincture of cantharides the power of retaining the urine was always restored *for the time.* This power would cease, and again be restored, on suspending or repeating the medicine. It is obvious that the cantharides acted through the segment of the excito-motory system left below the division of the spinal marrow." (p. 152.)

Dr. Hall subsequently directs attention to another important class of phenomena, in which the influence of the spinal system on the functions of organic life is remarkably displayed. "It is almost certain [the recent experiments of Valentin appear to have satisfactorily proved—see the first article in our present Number] that the gall-ducts, the ureters, and other excretory canals, are endowed with both incident and excitant, and with reflex and motor nerves. The passage of a biliary or urinary calculus excites vomiting; exposure to cold, a loaded intestine, certain passions, and in infants mere dentition, will, on the other hand, arrest the flow of bile, and induce icterus." (p. 157.)

The remainder of Dr. Hall's second memoir is occupied with an enquiry into the *retrograde* action, as he terms it, of stimuli acting on the spinal system of nerves. By this he wishes to generalize those phenomena, of no unfrequent occurrence, in which a stimulus conveyed to the lower part of the spinal cord, acts upon parts receiving their nerves from its upper segments. Now upon this we shall only remark that the fact of spasmodic actions being produced in almost every part of the body by excitants applied to parts below as well as above, has long been known to pathologists; and we do not see that, except in referring these actions, with others of a spasmodic nature, to his true spinal system, Dr. Hall has contributed anything to their explanation. To ourselves the matter appears quite simple. In the *true spinal cord* there is no such thing as *direct* and *retrograde*—terms which only apply to the course of that portion of it which consists of prolongations of the cerebral fibres. The true spinal cord may be regarded as a series of ganglionic centres belonging to the several segments of the body, and connected to each other by numerous sets of fibres passing in every direction. Nothing that we yet know of the course of these fibres explains why a stimulus transmitted to the upper part of the spinal cord should produce motion in the organs that receive nerves from the lower; and when this is understood we feel no doubt that similar connexions will be found to account for the converse phenomena, termed *retrograde* by Dr. Hall.

This memoir is concluded by a summary of inferences, the most important of which we have already interwoven in our account of it; and we shall therefore refer those who are desirous of following out the subject to the paper itself.

Dr. Hall commences his second memoir by pointing to what he considers the three causes or principles of muscular motion in the animal economy—*volition*, *emotion*, and the direct and reflex actions of the *vis nervosa.* In this enumeration we cannot but think there is some confusion: if the *vis nervosa* of Haller be intended (as Dr. M. Hall has constantly professed), this is alike the means by which volition, emotion, and simple reflex action produce muscular movement. No physiologist ever speaks of a volition or an emotion being transmitted along a nerve; these being mental conditions which perform the part of a stimulus applied to some part of the incident system of nerves, in producing a motor impulse, or action of the *vis nervosa* (if Dr. Hall likes the phrase), which being propagated through the efferent trunks produces muscular action. The question between us is evidently one more of words than of things; but if it be an object to conform to the Hallerian phraseology, we must again affirm that Dr. M. Hall, in separating *vis nervosa* from the influence of volition, makes a wrong use of the former term.

"Volition," remarks Dr. Hall, "has a constant influence over some of the muscular actions of which we are almost unconscious, and which we only discover by carefully observing the effects of its subtraction." The acts of respiration, for example, originate in the reflex function of the spinal cord; but are regulated and rendered equable by this silent but constant influence, as is shown by their laborious and irregular character during great attention, profound sleep, coma, &c. The position of the body and every action of locomotion involve the constant and almost equally unconscious influence of volition; yet the movements of many animals, especially birds and insects, are performed after the removal of the cerebrum, with a degree of energy which shows that the will rather guides and controls than immediately stimulates them. Hence, observes Dr. Hall, we may account in some degree for the long flights of birds in migration; since the actions of their wings partake of the character of those of respiration, and others belonging to the true spinal system, which are performed without fatigue.

The influence of emotion is often still more important, and still less observed. It is manifested in the movements of *expression;* also in the spasm of some muscles, and the relaxation of others; in the contractions of the heart and arteries; the peristaltic movements of the alimentary canal; probably in the similar movements of the efferent ducts of glands, which greatly modify the secretion; and not unfrequently in movements of parts which are paralysed to volition. The organs influenced by emotion are so completely those which are most governed by the spinal system, that we cannot hesitate in agreeing with Dr. Hall in regarding this as its channel; which view is, in fact, no more than an extension of that of Sir C. Bell, who considered the movements of expression as belonging to his respiratory system. But Dr. Hall does not advert to the fact that *emotions* are generally *excited* through the *special* senses, which do not belong to the spinal but to the cerebral system; we cannot, therefore, avoid regarding them as having a distinct centre, though they may use the nerves of the spinal and ganglionic systems as the instruments of their operation. The researches of Valentin and others have made the source of the motory powers of the ganglionic system now quite evident; its motor and sensory fibres being entirely derived from the spinal nerves. There is, as Dr. Hall justly remarks, a near connexion between emotion and hysteria, which is doubtless very much a disease of emotion; the same organs, the same functions, are affected; and in both cases the will has a considerable power of controlling the movements which would otherwise involuntarily take place.

In quiet sleep we have the absence of all volition and emotion, and of their effects; these all return during dreaming and on awaking. The

*first* sleep especially, and the transition from sleeping to waking, are circumstances peculiarly connected with certain diseases of the nervous system: thus the singular frequency of the attacks of the croup-like convulsion of infants late in the evening, and of epilepsy in adults on awaking in the morning. These are, however, only hinted at by Dr. Hall, who justly observes that " to trace these principles of action in their healthy and morbid relations must be very important; but this task can only be accomplished by long-continued observation, and in some cases by numerical deduction."

With a view of illustrating these opinions, Dr. Hall again refers to the phenomena of those nervous diseases, in which one part of the nervous centres is severely affected almost or altogether to the exclusion of the other. Pure and uncomplicated hemiplegia dissects and severs, as it were, the cerebral from the true spinal system, volition from emotion and the reflex action. The power of voluntary motion of one side is suspended; but the action of the sphincters, the respiratory movements, and other operations of the true spinal system remain unimpaired; and the arm is often remarkably affected by mental emotion. Hence Dr. Hall concludes that the seat of emotion must be below the disease, probably in the medulla oblongata; and that the fibres which conduct its influence do not decussate. On the other hand, in tetanus the cerebrum is unaffected, but the whole spinal system is deranged. The intellect is serene; but the excito-motor power is augmented, and the actions of the muscular system (especially of those parts of it concerned in the ordinary associated movements of respiration, deglutition, defecation, &c.) are morbidly violent; and any sudden or startling noise, or any external impression, as a sudden jar, the contact of cold air, &c., are attended by the most painful and agonizing exasperation of the symptoms. " Such indeed," remarks Dr. Hall, " is the baneful influence of these various excitements, that I am persuaded that the very same treatment of tetanus may be successful or unsuccessful, according as we carefully avoid or admit the influence of emotion and external stimuli. Bearing this fact in our mind, the patient should be kept as free as possible from the intrusion of visitors, and should be carefully surrounded by an atmosphere of uniformly elevated temperature charged with moisture, every draught of wind, and all exposure of the cutaneous surface being cautiously avoided. We all remember the case in which the sudden plunge into a cold bath proved fatal. Other but less severe agencies of the same kind may prove injurious, though in a less terrible degree. Stillness and even darkness are essential to the safety of the patient." Although experience alone might have established the importance of these therapeutic rules, they derive additional weight from the knowledge of the manner of their influence. In traumatic tetanus, an excitant conveyed to some particular spot in the spinal cord, affects the motor nerves which pass off above as well as below it, constituting an example of what Dr. Hall terms the retrograde as well as the direct action of the spinal system.

In reference to the influence of emotion on the nervous system, Dr. Hall adverts to the fact that the irregular movements of chorea and incipient paralysis agitans subside during *quiet* sleep, but return during the agitation of dreaming; and also alludes to the well-known influence of emotion in aggravating stammering, the true idea of which irregularity is, he considers, that of certain voluntary acts impeded and modified by emotion. We should rather say that there is an original deficiency of power to control and combine the several movements; and that this is aggravated by emotion.

Dr. Hall might have further illustrated his views by referring to cases (of which there is, we believe, more than one on record) in which the power of expression (an emotional act) has been lost along with the power of performing other sympathetic or excited movements, as those of respiration, whilst the muscles have remained completely under the control of volition. We feel no doubt that the practical applications of the discoveries which the last few years have witnessed in nervous physiology are as yet only in their infancy; and we would strongly urge our readers to do their best towards their improvement and extension, by applying themselves to the more careful discrimination of the phenomena of disease than has yet been customary; a discrimination which can only be exercised where the mind is under the guidance of sound physiological knowledge.

XI. Mr. H. B. Jones next gives a short paper "*On the presence of sulphur in cystic oxide calculus.*"

XII. Mr. Arnott, gives the case of a "*Large osseous tumour of the uterus,*" which weighed five pounds, yet occasioned no inconvenience during life.

XIII. In the next paper, by Mr. Dalrymple, "*On the rapid organization of lymph in cachexia,*" an attempt is made to show "that abnormal effusions from capillary vessels, without direct rupture of their coats, are more speedily and completely organized with vessels capable of being permeated by our minute injections, in those feeble and depressed conditions which we denominate cachectic than in the more vigorous and plethoric, where inflammations are more acute in the outset, and pass through more speedy and determined changes." In support of this view Mr. Dalrymple refers to the rapid organization of fibrine in cases of iritis occurring in habits very much debilitated from syphilis, or mercury, or other causes; to the progress of malignant disease of the eye in children, which, as in the case mentioned, appears to be influenced in its growth by the debilitated condition of the general system; to the vascularity of the lining membrane of some abscesses which were formed around a diseased knee-joint of very long standing, and where there was no vigour of constitution; and to an instance of scurvy, in which a quantity of blood effused beneath the periosteum of the tibia, was shown by injection to be most intimately permeated by vessels.

"The subject," says Mr. Dalrymple, "here briefly entered into is of vast extent, and appears to me interesting as a physiological enquiry; at the same time, it cannot be denied that in practice it would become important should future enquiry determine that the organizable materials of the blood become sooner and more completely organized in the debilitated and cachectic subject than in healthier and more robust constitutions."

The subject is certainly one of much importance, and Mr. Darymple has done a service by thus calling attention to it. But we do not think that the facts which he has brought forward go far enough to establish the great rapidity of the organization of lymph in cachectic subjects. Before any precise inference can be drawn in such cases, we require to have the cachectic condition defined, or as nearly as possible defined, and this compared with the opposite state; also distinct evidence of the time which this or that product has occupied in becoming organized—such, for instance, as the lining membranes of abscesses mentioned, and the effused blood, and these compared with other abscesses formed in individuals not cachectic, and other quantities of blood effused (and not absorbed, another item in the question) in subjects not scorbutic. We make these observations only as hints and as difficulties, but still considering them important in the enquiry which has been entered into by Mr. Dalrymple, and which we hope he will pursue.

XIV. Mr. Stafford has related an instance of "*Recovery from cut throat, in which both the larynx and thorax were extensively opened.*"

XV. The next communication is from Mr. Bloxam, "*On the structure of the human placenta, and its connexion with the uterus.*" It is carefully executed, and illustrated by drawings, but scarcely a subject for notice in the present analysis.

XVI. Mr. Solly has given the "*Termination of the case of dry gangrene,*" mentioned in vol. xxii.

XVII. There then follow some "*Observations on injuries of joints, and their treatment.*" By Mr. R. Alcock.

Mr. Rutherford Alcock's paper is most carefully composed, and the facts and inferences from facts which it contains are apparently derived from extensive experience as a military surgeon. He is guided by safe principles in the views which it is his endeavour to establish, and we recommend the whole paper as worthy of attentive perusal, especially by those who are intended to act as military surgeons. Speaking of the danger of hasty generalization from a few facts, he observes, and the caution cannot be too often repeated:

"In the class of injuries under consideration this danger is especially evident. Many are the extraordinary and most unlooked-for successes attending the treatment of forlorn cases of injured joints. Were general rules or principles of treatment to be founded on these cases, which are but units among thousands giving contrary results, and were no reference made to those greater numbers which enlarged experience shows must perish in vain attempts to save limbs, an immense sacrifice of life and increase of human suffering would inevitably follow." (p. 245.)

The following remark respecting the saving of a limb it is well to remember:

"By a limb saved, I do not mean one with the wounds healed, having, nevertheless, the extremity contracted, bent, motionless, or otherwise useless; cases which by a loose kind of phraseology are often termed 'limbs saved.' The object of saving a limb is that it may be useful. If this is not the result, the member, by merely hanging to the body of the patient, is lost in my estimation as truly as if amputated, but with the additional circumstance of being converted into a source of misery to the sufferer, an impediment to the free motion of the rest of the body, and often a cause of irremediable ill health. Such cases I hold to be among the worst specimens of bad and injudicious surgery." (p. 246.)

In speaking of the excision of the ends of bones when injured by shot, it is considered that this operation is most applicable to the shoulder, elbow, wrist, and ankle; that it is scarcely applicable to the hip and knee; and that it is most likely to be useful where the head of the humerus alone is implicated, and that by a musket-ball.

The results at which Mr. Alcock has arrived are the consequences of a careful analysis of about 100 cases of severe injury to joints, the notes of which he had taken care. Some of these results are presented in tables, these tables containing only the accounts of gun-shot wounds. And it is satisfactory that the author is fully alive to the mistakes into which statistics may, if not very carefully managed, lead us. "If," he says, "*all* the cases of a given period be included, they form sufficient grounds for just conclusions; but if one case be omitted the whole return is falsified: it may be a death, or a cure, or an amputation; but whatever the termination, its omission alters the legitimate conclusion." We shall endeavour to present to our readers such a brief analysis of Mr. Alcock's paper as may not be without practical interest.

All the injuries of joints are classed under three heads: 1. Where the limb may be saved, and where it should be a principle of practice to attempt this. 2. Doubtful cases, where diagnosis and treatment are difficult, where each case requires its separate consideration; but as they ultimately must require more or less protracted treatment, the same principles adopted for the first apply. 3. Where immediate amputation is required.

In lacerated or incised wounds penetrating the capsule of joints, Mr. Alcock does not think it of so much importance as it is mostly supposed to be, to exclude the air. Light dressings without any compression, cold applications, succeeded if agreeable to the patient's feelings by warm-water dressings:

"Or if the joint has assumed a puffy, swelled, and unhealthy appearance, a state often to be traced to the injudicious use of poultices, a more tonic and stimulating mode of dressing will generally cause improvement. Of this kind of dressing, the best seem to me either a decoction of aromatic herbs with the addition of a little wine, or warm camphorated or sweetened wine, which has not been freely adulterated with bad brandy, as are generally most of the wines consumed in England. Such dressing is frequently employed in the rest of Europe, and I have no hesitation in saying that I have seen the happiest effects from its use where more emollient applications, such as poultices, certainly did not arrest, but, on the contrary, appeared to promote the 'engorgement' of the limb." (p. 267.)

Pus must be freely evacuated when formed. Great quiet is long necessary. Some motion may be acquired if anchylosis be partial, and some force may be used to effect this; but if anchylosis be complete no attempts of this kind should be made. We would call attention to this last observation respecting the attempts which may be made to restore motion of anchylosed joints, in connexion not alone with gun-shot wounds but also in reference to the attempts which are now so frequently made to restore the proper position of deformed joints. There is sometimes great carelessness in the choice of cases for these operations, and we have seen partial or nearly complete anchylosis with contracted ham-strings following injury of the joint, in which the attempt to restore mobility disturbed the anchylosis and brought back the morbid action in the joint.

Mr. Alcock has given an analysis of the tables to which we have already referred. The following heads are those under which this analysis is given :

"1. Their proportionate numbers in relation to other classes of injuries, and of the articulations with each other. 2. Mortality, absolutely and relatively; number of amputations to which these injuries give rise, and proportionate number in different periods. 3. Causes of mortality, with regard to the whole number of deaths and to the number of deaths from each articulation, considered in relation to amputation at the three periods, primary, intermediary, and secondary, and to cases treated without amputation. 4. Influence of external and collateral circumstances." (p. 270.)

We have quoted the above arrangement that our readers may judge of the scope of Mr. Alcock's observations on the subject; from what follows we shall but extract such matter as is of practical or special interest, leaving a reference to the paper itself for the lovers of numerical calculations and detail.

The proportion of injured joints was found to be much less than Mr. Alcock supposed before he took the trouble to keep an accurate account of them; and of all joints none is so frequently injured as the knee. The mortality in all the cases of injured joints is very large indeed, taking into account deaths proceeding from the immediate consequence of injury to the joint itself, and injury proceeding from disease extending to the joint from the part primarily affected.

Mr. Alcock has taken pains to state general results in as clear a manner as possible, because the tendency of many of his observations is to lead to the saving rather than to the amputation of injured limbs. The following observations are important:

"Where the joints were directly involved, the number *treated*, that is to say, in which primary amputation was not performed, amounting to forty-four, present a mortality of twenty-five, considerably more than one half, whereas the primary amputations cause a loss of only one third, although naturally performed for the very worst injuries : and while twelve only were cured without loss of limb, eighteen died in the vain attempt to save, without for the most part offering any fair opportunity of remedying the evil by intermediary or secondary operation. Of the intermediary and secondary amputations, where treatment failing to save the limb amputation offered the only ground of hope for life, seven died out of fourteen, amounting to one half; but of the secondary amputations, properly so called, a fraction less than one half were lost, five in eleven. These cases form the forlorn hopes of surgery; all saved are snatched from nearly certain death." (p. 275.)

Injuries of the hip are commonly fatal. Of injuries of the shoulder the following satisfactory observations are made:

"The shoulder is rarely implicated directly by injury without a subsequent operation, amputation, or excision of the head of the humerus being required. In eleven,....only two were cured without amputation; seven amputations were performed, six primary and one intermediary: the latter was unfortunate in its result; all the primary recovered." (p. 277.)

We have already stated that injuries of the knee are most frequent, and excepting those of the hip, most fatal to life. Of 35 cases of injury of the knee, 22 died, and of the remaining 13 who were saved, 8 lost their legs. Injuries of the elbow are next in order of frequency; their fatality is in the proportion of 5 to 19. Frequently, it is remarked, that although divers injuries happen in the neighbourhood of joints, comminuting the bones and producing extensive ill effects, the joints themselves are comparatively little affected, but are useful afterwards. Of 43 fatal cases, the deaths are thus arranged: 23 died under treatment for the original injury; 4 after intermediary amputation; 5 after secondary amputation; 11 after primary amputation. Of the first 23, half died, chiefly from hectic; the other half "from supervening irregular actions, such as mortification, delirium tremens, tetanus, affections of the chest complicating the hectic state; from accidental occurrences, such as secondary hemorrhage, and from other complicating wounds." Mr. Alcock has not found in his own experience that in these cases there is any particular tendency to purulent depositions in other organs or parts of the body; and when these have taken place it has been in the majority of cases where amputation has been performed after injuries, where "the two shocks of the injury and the operation combine to produce this fatal effect." Mr. Alcock's view of this matter is different from that which has been put forward, that purulent depositions in distant organs are distinctive of secondary amputations. It is probable that other considerations besides those admitted by Mr. Alcock are of great importance in relation to any decision on this point. The determination of the connexion of purulent depositions in various parts of the body subsequent to injuries and operations, is perhaps the determination of the circumstances altogether the most favorable to the production of phlebitis or whatever may be the cause of these depositions. To take any number of similarly injured joints, in men differently circumstanced as to climate, season, comforts or the contrary, epidemic or other influences, and then to make the fact of primary or secondary amputation the one essential fact in determining the connexion of purulent deposition and primary or secondary amputation, is, in our view of the subject, entirely erroneous. It is characteristic of the uncertainty attaching to all attempts at accurate deductions from the majority of facts with which it is in our power to become acquainted, that they will not bear the strict examination which we should wish to be able to apply to them, but at the best allow us to conclude only from the greater amount of probability. On the recapitulation or summary* of the facts related, Mr. Alcock remarks:

"The chief danger and cause of death in cases treated to the end without operation is hectic fever, and a variety of accidental or irregular complications, such as secondary hemorrhage, epidemics, erysipelas, gangrene, &c. combined, form the remaining half. The cases in which amputation is performed in the first instance, with fatal result, present a very different cause of mortality: the chief agent being purulent disease of lungs or liver, and occasionally inflammatory affections of the lungs or pleura. Fevers, irritative or bilious, destroy more than one third. The deaths after intermediary amputations are chiefly caused by febrile action, irritative or bilious, and in secondary amputations, the shock of the operations, hectic and some accidental complications carry off the patients already much reduced by suffering and the long continuance of wasting discharge. The results of secondary amputations when fatal, and their causes of

* We wish Mr. Alcock would not employ the Gallic barbarism *resumé*, when his own language, which he knows how to use so well, supplies so unexceptionable substitutes as the words in the text.

mortality, are in some degree assimilated to those predominant in cases treated to the end without operation." (p. 287.)

After some remarks on the influence of favorable and unfavorable circumstances on the class of injuries here treated of, the paper terminates by a classification of the kinds of injury in wounds of the articulations. In this classification are noticed some important points and rules of practice, which we regret not to be able to refer to at greater length.

We cannot concur with Mr. Alcock in supposing that some of his conclusions from cases related are anything more than mere hints to the proper mode of practice; there is such a want of uniformity in the immediate and remote effects of apparently similar injuries, that it would require a large class of cases to derive anything like a rule which should be a satisfactory guide. These remarks we would apply to the conclusion drawn from cases 4, 5, 6, as well as to other conclusions which can only be regarded, as we have said, as hints to proper treatment; at the same time they are valuable hints, and such as we shall do well to study and consider. All the cases related by Mr. Alcock are well worthy of attention, and some especially, as giving reason to hope that many limbs, the joints of which have been extensively injured, may be saved and rendered serviceable, under circumstances which would generally be regarded as demanding their removal. We cannot conclude our notice of Mr. Alcock's paper without most especially recommending it to our readers, and without expressing our regret that our notice of it is necessarily so limited.

Our notice of the papers in this volume has already extended so far that we can only give the title of the remaining communications.

XVIII. The first is a very elaborate and valuable one, "*On aneurisms, and especially spontaneous varicose aneurisms of the ascending aorta and sinuses of Valsalva, with cases;*" by John Thurnam, Esq.; and which we recommend to the notice of our readers.

XIX. Mr. Curling has described a "*Rare species of hydatid* (*the echinococcus hominis*) *found in the human liver.*"

XX. By a series of experiments on animals, Mr. R. H. Meade has shown the similarity between the processes of repairing fractures in long and flat bones.

XXI. Mr. Wickham next relates a case, in which for aneurism of the arteria innominata he tied both the carotid and subclavian arteries, and with some apparent temporary benefit. The patient, however, died shortly afterwards from rupture of the aneurismal sack.

XXII. The volume terminates by a case communicated by Mr. Lever, showing the advantage of evacuating, through an incision made in the vagina, encysted tumours occupying the pelvis, and acting as impediments to parturition.

